# Effects of Bisphosphonate Treatment on Circulating Lipid and Glucose Levels in Patients with Metabolic Bone Disorders

**DOI:** 10.1007/s00223-021-00811-w

**Published:** 2021-02-09

**Authors:** Gabriella Iannuzzo, Gianpaolo De Filippo, Daniela Merlotti, Veronica Abate, Alessio Buonaiuto, Marco Evangelista, Marco Gentile, Alfonso Giaquinto, Tommaso Picchioni, Matteo Nicola Dario Di Minno, Pasquale Strazzullo, Luigi Gennari, Domenico Rendina

**Affiliations:** 1 Department of Clinical Medicine and Surgery, Federico II University, Naples, Italy, 5, Via Pansini, 80131 Naples, Italy; 2 Assistance Publique-Hôpitaux de Paris, Hôpital Universitaire Robert-Debré, Service d’Endocrinologie et Diabétologie Pédiatrique, Paris, France; 3 Department of Medicine, Surgery and Neurosciences, University of Siena, Siena, Italy; 4 Department of Translational Medical Sciences, Federico II University, Naples, Italy

**Keywords:** Paget’s disease of bone, Osteoporosis, Bisphosphonate, Glucose, Cholesterol, Triglycerides

## Abstract

Bisphosphonates are the first-choice treatment of osteoporosis and Paget’s disease of bone. Among the bisphosphonates, the non-amino-bisphosphonates, such as clodronic acid, are intracellular converted into toxic analogues of ATP and induce cellular apoptosis whereas the amino-bisphosphonates, such as zoledronic acid, inhibit the farnesyl-diphosphate-synthase, an enzyme of the mevalonate pathway. This pathway regulates cholesterol and glucose homeostasis and is a target for statins. In this retrospective cohort study, we evaluated the effects of an intravenous infusion of zoledronic acid (5 mg) or clodronic acid (1500 mg) on blood lipid (i.e. total cholesterol, low-density lipoprotein-cholesterol, high-density lipoprotein-cholesterol and triglycerides) and glucose levels in patients with osteoporosis and Paget’s disease of bone. All patients were evaluated before, 1 and 6 months after bisphosphonate treatment. Pagetic and osteoporotic patients treated with zoledronic acid showed a significant reduction in glucose and atherogenic lipids during follow-up whereas these phenomena were not observed after clodronic treatment. The effect on circulating lipid levels was similar in naïve and re-treated Pagetic patients. Zoledronic acid treatment was associated with a reduction in blood glucose and atherogenic lipids in patients with metabolic bone disorders. The extent of change was similar to that obtained with the regular assumption of a low-intensity statin. Further studies are warranted to better evaluate the clinical implications of these observations.

**Supplementary Information:**

The online version of this article (10.1007/s00223-021-00811-w) contains supplementary material, which is available to authorized users.

## Introduction

Atherosclerosis is a leading cause of cardiovascular morbidity and mortality in Western countries and dyslipidemia is one of the most significant risk factors [1]. Recently, some studies have focused on a possible relationship between metabolic bone disorders (MBDs), cardiovascular diseases (CVDs) and cardiovascular risk factors, primarily dyslipidemia, also considering that the incidence and prevalence of MBDs and CVDs are very high in the adult population worldwide [2, 3]. Osteoporosis (Op) is a MBD characterized by low bone mass and structural deterioration of bone tissue, with a consequent increase in bone fragility and susceptibility to fracture. Op affects approximately 200 million people and leads to nearly 9 million fractures annually worldwide [4]. Paget’s disease of bone (PDB) is the second most common MBD after Op worldwide, although marked ethnic and geographical variations in its prevalence has been observed [5–9]. PDB is characterized by increased and disorganized bone turnover, which affects one or more skeletal sites, i.e. monostotic and polyostotic PDB, respectively. The skeletal sites involved by PDB show pathognomonic radiological and scintigraphic changes [9]. Bisphosphonates (BPs) are effective antiresorptive agents currently available for the treatment of several MBDs including Op and PDB [10]. BPs are synthetic, non-hydrolysable analogues of pyrophosphate. There are two different classes of BPs: non-amino-BPs (nN-BPs) and amino-BPs (N-BPs). The nN-BPs, such as clodronic acid (Clo), are converted intracellularly to methylene-containing analogues of ATP (AppCCl2p). The accumulation of AppCCl2p in osteoclasts in vitro inhibits bone resorption by inducing osteoclast apoptosis. On the contrary, the N-BPs such as zoledronic acid (Zol) are not metabolized into toxic intracellular analogues of ATP, but act by inhibiting farnesyl-diphosphate-synthase, a key enzyme of the mevalonate pathway. N-BPs are several orders of magnitude more potent than nN-BPs at inhibiting bone resorption in vivo [10, 11]. The mevalonate pathway inhibited by N-BPs has a central role in cholesterol (Chol) biosynthesis and in glucose homeostasis and is a well-known molecular target also for statins, the first-choice treatment for dyslipidemia [12]. In consideration of the above, the objective of this retrospective cohort study was to evaluate if BP treatment affects lipid and glucose blood levels in patients with PDB treated with Zol or Clo. We also analyzed data collected from patients with complicated Op treated with Zol.

## Materials and Methods

This retrospective cohort study was performed analyzing the medical records of all consecutive Caucasian patients with PDB [International Classification of Diseases (ICD)-9 code 731.0] and with complicated Op (ICD9 codes 73310-9) referring from January 01, 2009, to December 31, 2018, at the Units of Bone and Mineral Disorders of Siena and Naples, two main national centers for the diagnosis and treatment of MBDs which used a unique approach for clinical classification and management of MBDs and their comorbidities [13, 14]. On their first admission, all patients were informed that (i) their personal data, collected as part of administrative management and hospital care, could be used for health research purposes, under the responsibility of the Federico II University of Naples and of Siena University; (ii) they could withdraw their consent to the use of personal data without providing further explanation at any time and without medical assistance being affected. This, in the form of written informed consent, was obtained from each patient or subject involved in this study.

Data collection was performed from July 01, 2019, to December 31, 2019. The PDB and Op diagnoses were based on published criteria [9, 15]. Only medical records of patients treated with Zol 5 mg intravenously (i.v.) or Clo 1500 mg i.v. were selected for this study [16, 17], to compare N-BP to nN-BP regimens. The BP treatment was administered after supplementation of cholecalciferol and/or calcium in all cases (http://www.agenziafarmaco.gov.it/content/nota-79) [18]. Cholecalciferol supplementation and an adequate calcium dietary intake were also prescribed after BP treatment (http://www.agenziafarmaco.gov.it/content/nota-79) [18, 19]. According to a pre-established collection schedule, the following data were extracted from selected medical records: age, sex, body mass index (BMI), data of infusion, type of drug, glycaemia (sGlu), serum calcium (sCa), total Chol (tChol), low-density lipoproteins-Chol (LDL), high-density lipoprotein-Chol (HDL), triglycerides (TG), 25OH-vitamin D (25OHD), total alkaline phosphatase (ALP), and creatinine (sCrea) levels. These data were collected at the time of BP infusion (T0), after 1 (T1) and 6 months (T6) of treatment. All parameters were expressed according to Système International d’unités excluding ALP serum levels which, in absence of universally accepted reference ranges, were expressed as percentage versus the upper limit of the reference range. Exclusion criteria comprised the following: age lower than 40 years, primary or secondary bone cancers [20, 21], malabsorption syndromes, rheumatoid arthritis, long-term immobilization, moderate to severe chronic kidney disease (estimated glomerular filtration rate (eGFR) ≤ 60 ml/min/1.73 m^2^ [22]), hypothyroidism [23], hyperthyroidism [23], primary hyperparathyroidism [24], hypoparathyroidism, Cushing’s syndrome, chronic liver disease, prostate cancer, pituitary tumors, surgical history of terminal ileal resection, gastrectomy or small bowel bypass, orchiectomy, eating disorders, alcoholism, diabetes mellitus, use of statins, anti-diabetes drugs, gonadotropin-releasing hormone agonist, glucocorticoids, anticonvulsants, heparin, vitamin A, cytotoxic agents and antiandrogens, and finally subjects with incomplete data collection. The study was approved by the Federico II Ethical Committee.

### Statistical Analysis

All statistical analyses were performed using the IBM SPSS Statistics, version 23 (International Business Machines Corporation, Inc., Armonk, NY, USA). Data are expressed as means ± standard deviation for continuous variables and as absolute; percentage values for discrete variables. Contingency table *χ*^2^ tests and analysis of variance (with Bonferroni correction for multiple comparisons) were used to test for between-group differences in non-parametric and parametric variables, respectively. Differences from baseline after BP treatment were estimated using the Student’s *t* test or the Wilcoxon test for variables with normal or not-normal distribution, respectively. A *p* value < 0.05 was considered statistically significant.

## Results

**Fig. 1:**
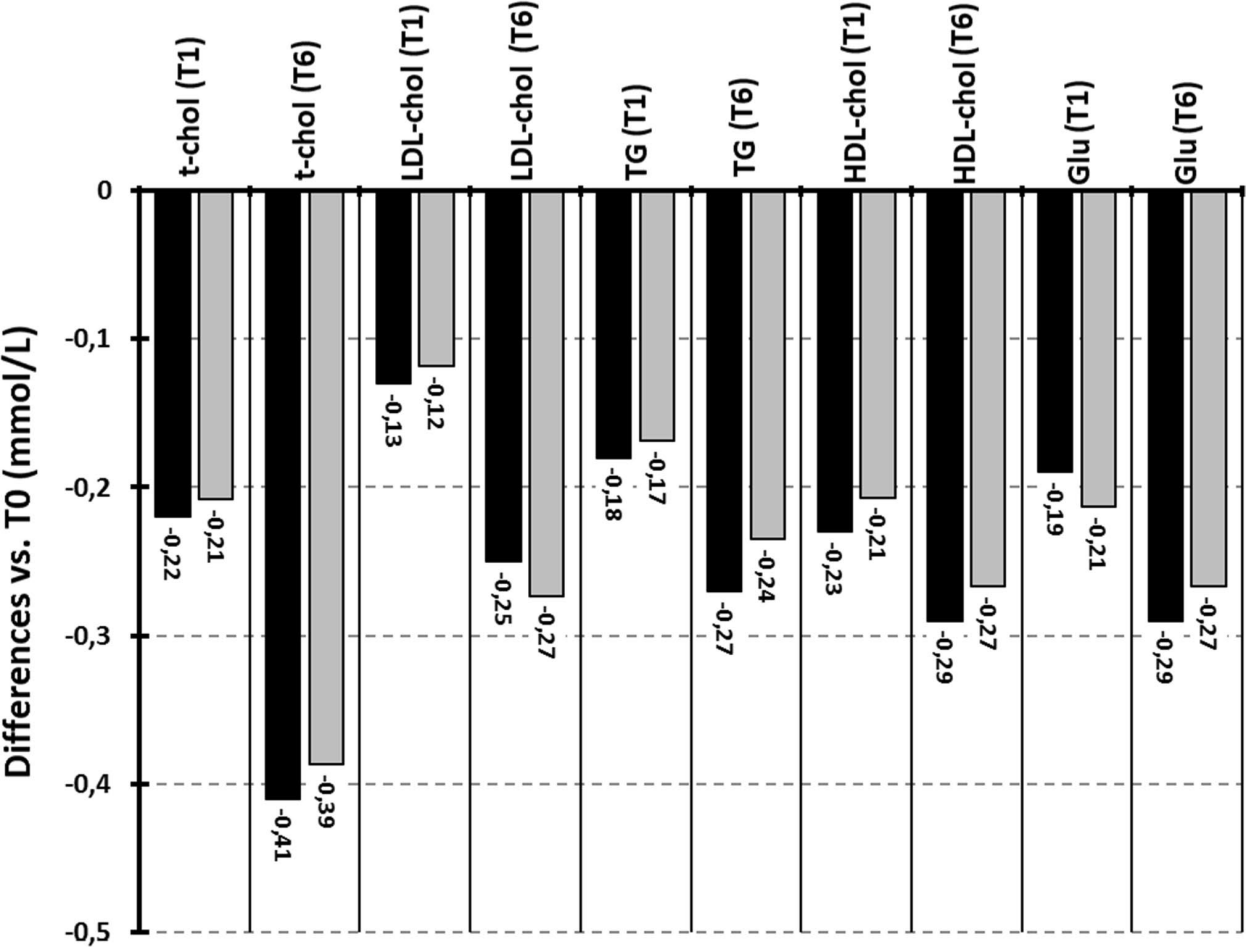
Differences from baseline in blood lipids and glucose levels in patients with Paget’s disease of bone treated for the first time with bisphosphonate (black columns) or in PDB patients previously treated with bisphosphonate (gray columns). Biochemical parameters were measured 1 month and 6 months after zoledronic acid infusion. *t-chol*(*T1*) serum total cholesterol 1 month after infusion, *t-chol*(*T6*) serum total cholesterol 6 months after infusion, *LDL-chol*(*T1*) low-density lipoprotein-cholesterol 1 month after infusion, *LDL-chol*(*T6*) low-density lipoprotein-cholesterol 6 months after infusion, *TG*(*T1*) serum triglycerides 1 month after infusion, *TG*(*T6*) serum triglycerides 6 months after infusion, *HDL-chol*(*T1*) high-density lipoprotein-cholesterol 1 month after infusion, *HDL-chol*(*T6*) high-density lipoprotein-cholesterol 6 months after infusion, *GLU-T1* glucose 1 month after infusion, *GLU-T6* glucose 6 months after infusion

According to inclusion and exclusion criteria previously exposed, the medical records of 87 PDB patients treated with Zol (PDB-Zol; men:women 52:35, mean age 69.4 ± 9.5 years; body mass index 26.4 ± 2.0 kg/m^2^, monostotic:polyostotic PDB 41:48), 69 PDB patients treated with Clo (PDB-Clo; men:women 38:31, mean age 66.7 ± 10.1 years; body mass index 26.1 ± 2.0 kg/m^2^, monostotic:polyostotic PDB 31:38) and 47 OP patients treated with Zol (Op-Zol; men:women 5:42, mean age 66.9 ± 10.1 years; body mass index 25.8 ± 1.7 kg/m^2^) were selected. Biochemical parameters of the three study cohorts before BP treatment (T0) as well as 1 month (T1) and 6 months (T6) after BP treatment are reported in Table 1. At T0, Op-Zol patients showed ALP levels significantly lower compared to both PDB-Zol and PDB-Clo (*p* < 0.01, analysis of variance with Bonferroni correction). No additional significant differences were observed between the three study cohorts at T0 (*p* > 0.05 in all cases, analysis of variance with Bonferroni correction). As expected, in PDB-Zol and PDB-Clo, ALP serum levels at T1 and T6 were significantly lower compared to T0 (*p* < 0.05 in all cases, *t* test for paired samples). At T1, PDB-Zol showed serum levels of sGlu, tCol, and LDL significantly lower compared to T0 levels (*p* values < 0.05 in all cases, *t* test for paired samples). At T6, PDB-Zol showed serum levels of sGlu, tCol, Try and LDL significantly lower compared to T0 levels (*p* values < 0.05 in all cases, *t* test for paired samples). Also OP-Zol showed serum levels of sGlu, tCol, and LDL significantly lower at T1 compared to T0 (*p* < 0.05 in all cases, *t* test for paired samples) and serum levels of sGlu, tCol, Try, and LDL at T6 compared to T0 (*p* < 0.05 in all cases, *t* test for paired samples). Conversely, PDB-Clo showed no significant variation in serum levels of sGlu, tCol, Try, and LDL at T1 and T6 compared with T0 (*p* values > 0.05 in all cases, *t* test for paired samples). The remaining biochemical parameters reported in Table 1 were not significantly different at T1 and T6 compared to T0 in the three study cohorts (*p* < 0.05 in all cases, *t* test for paired samples). As shown in Fig. 1, in PDB -Zol, the changes in tCol, LDL, Try, HDL, and sGlu levels were similar in patients receiving treatment for the first time (naïve PDB-Zol, *n* = 12, mean age 65.9 ± 9.8 years; M:F 7:5; BMI 25.4 ± 1.92 kg/m^2^) and in those previously treated with BPs (re-treated PDB-Zol, *n* = 75, mean age 69.9 ± 9.3 years; M:F 45:30; BMI 26.6 ± 2.10 kg/m^2^). The absolute and percentage variations (*Δ*) of each biochemical parameter measured in the three study cohorts at T1 and T6 compared to T0 are reported in Supplemental Table 1. The changes in circulating levels of sGlu, tCol, Try, and LDL observed at T1 and T6 were similar in magnitude between PDB-Zol and Op-Zol (*p* > 0.05 in all cases, analysis of variance). Moreover, we observed a direct relationship between ΔsGlu and ΔLDL-chol in Zol-treated patients (*r* = 0.245, *p* = 0.02) whereas no relationship was observed between Δ25OHD and ΔGlu (*r* =  − 0.22, *p* = 0.15), ΔLDL (*r* =  − 0.18, *p* = 0.45), ΔtChol (*r* =  − 0.20, *p* = 0.41), ΔHDL (*r* =  − 0.19, *p* = 0.42), and ΔTry (*r* =  − 0.26, *p* = 0.10).

**Table 1:** Biochemical parameters of the three study cohorts before and after bisphosphonate treatment

	PDB-Zol			PDB-Clo			Op-Zol		
	T0	T1	T6	T0	T1	T6	T0	T1	T6
Crea (μmol/l)	79.6 ± 31.3	81.1 ± 32.1	80.3 ± 31.2	80.9 ± 29.2	82.9 ± 30.3	79.6 ± 30.1	80.9 ± 30.6	82.1 ± 31.2	79.9 ± 29.9
Glu (mmol/l)	5.19 ± 0.58	4.98 ± 0.54^a^	4.91 ± 0.52^a^	5.23 ± 0.52	5.41 ± 0.51	5.27 ± 0.54	5.25 ± 0.51	4.97 ± 0.51^a^	4.90 ± 0.49^a^
tCa (mmol/l)	2.37 ± 0.13	2.29 ± 0.12	2.32 ± 0.12	2.41 ± 0.14	2.29 ± 0.13	2.40 ± 0.13	2.34 ± 0.13	2.21 ± 0.12	2.32 ± 0.13
t-chol (mmol/l)	5.10 ± 1.06	4.89 ± 1.02^a^	4.71 ± 1.01^a^	5.29 ± 0.99	5.18 ± 1.06	5.23 ± 1.05	5.09 ± 1.02	4.86 ± 1.05^a^	4.72 ± 1.03^a^
HDL-chol (mmol/l)	1.32 ± 0.41	1.31 ± 0.42	1.31 ± 0.43	1.49 ± 0.41	1.39 ± 0.42	1.39 ± 0.41	1.51 ± 0.39	1.50 ± 0.41	1.50 ± 0.39
LDL-chol (mmol/l)	3.10 ± 0.96	2.98 ± 0.89^a^	2.83 ± 0.86^a^	3.10 ± 0.83	3.11 ± 0.82	3.16 ± 0.85	2.92 ± 0.81	2.76 ± 0.82^a^	2.66 ± 0.79^a^
TG (mmol/l)	1.49 ± 0.80	1.32 ± 0.82	1.25 ± 0.81^a^	1.53 ± 0.86	1.48 ± 0.82	1.50 ± 0.81	1.46 ± 0.78	1.31 ± 0.80	1.24 ± 0.80^a^
25OHD (nmol/l)	84.3 ± 18.7	82.4 ± 19.1	83.7 ± 17.8	89.1 ± 18.4	88.4 ± 18.1	87.1 ± 17.2	85.1 ± 17.6	83.4 ± 17.8	81.6 ± 16.9
ALP (%)	155 ± 90^b^	87 ± 32^a^	78 ± 27^a^	148 ± 84^b^	114 ± 58^a^	83 ± 36^a^	75 ± 22	67 ± 21	65 ± 26

Data are expressed as mean ± standard deviation

*PDB-Zol* patients with Paget’s disease of bone treated with zoledronic acid, *PDB-Clo* patients with Paget’s disease of bone treated with clodronic acid, *Op-Zol* patients with osteoporosis treated with zoledronic acid, *T0* before zoledronic or clodronic acid treatment, *T1* 1 month after zoledronic or clodronic acid treatment, *T6* 6 months after zoledronic or clodronic acid treatment, *Crea* creatinine, *Glu* glycemia, *tCa* total calcium, *t-chol* total cholesterol, *HDL-chol* high-density lipoprotein-cholesterol, *LDL-chol* low-density lipoprotein-cholesterol, *TG* triglycerides, *25OHD* 25OH-vitamin D, *ALP* total alkaline phosphatase

^a^Significantly different compared to T0 (*p* < 0.05; *t* test for paired samples)

^b^Significantly different compared to Op-Zol patients (*p* < 0.05; analysis of variance with Bonferroni correction)

## Discussion

The main finding of this study is that a single Zol 5 mg dose, used for the treatment of PDB or Op [9, 15–18], is associated with a simultaneous reduction in circulating glucose and atherogenic lipids levels in subjects without a personal history of diabetes and/or dyslipidemia. These effects were observed both at T1 as well as at T6. This pharmacological property seems to be characteristic of the Zol action on the mevalonate pathway. In fact, no change in circulating glucose and atherogenic lipids was observed in PDB patients treated with Clo, a nN-BP displaying a different mechanism of action [10, 11]. Since Zol and Clo regimens caused a similar reduction in serum ALP levels of PDB patients, it is likely that the observed reduction in the circulating levels of glucose and atherogenic lipids in these patients was not directly linked to the inhibition of abnormal bone turnover. Remarkably, the extent of change observed in circulating atherogenic lipids after Zol infusion is similar in magnitude to that obtained with the regular assumption of a low-intensity statin [25]. Our study results are in agreement with those previously reported in patients treated with Zol for malignancy [26], and strengthen the hypothesis of a link between the N-BP mechanism of action and energetic metabolism in patients with MBD, who are considered at increased risk of CVDs (particularly in case of increased bone turnover, such as PDB and postmenopausal Op) [27]. Indeed, MBD and CVDs may share common pathogenic pathways: in particular, several molecules (such as bone morphogenetic proteins, receptor activator of nuclear factor κB ligand, osteoprotegerin, matrix Gla protein and cathepsins), the parathyroid hormone–vitamin D–calcium–phosphate biological system, oxidized lipids and vitamin K seem implicated in both bone and cardiovascular metabolism [28–30]. In addition, experimental studies support a role of osteocytes in the regulation of lipid metabolism [31]. On clinical grounds, PDB is considered a risk factor for CVDs occurrence, based on the higher prevalence of vascular calcifications and cardiac dysfunction reported in PDB patients [32–35]. The observed reduction in circulating levels of atherogenic lipids and glucose by N-BPs seems to be a pharmacological property that is additive to the previously described capacity of N-BPs to reduce the accumulation of lipids in the atherosclerotic plaque and to prevent vascular calcifications [36–40].

Our results are also in agreement with those reported by Sing and colleagues showing that N-BP treatment is associated with a significant reduction in cardiovascular morbidity and mortality in patients with newly diagnosed hip fracture [41]. Other cohort studies have reported that alendronate, another N-BP, significantly decreases the risk of CV events in a dose-dependent fashion in patients with severe osteoporosis [42–44]. While there is evidence that vitamin D deficiency unfavorably affects glucose and lipid metabolism [29, 45, 46], the changes in glucose and atherogenic lipids observed in our study population upon N-BP treatment were not related to changes in 25OHD serum levels. In fact, all the patients enrolled in this study were treated with cholecalciferol before and after BP treatment, according to the criteria proposed to Italian Health System, to prevent acute-phase reaction and potentially dangerous electrolyte changes after N-BP treatment [18, 19]. Altogether, these epidemiological and experimental observations add further relevance to our study results, which nevertheless are affected by some limitations. The major limits of the study mainly include its retrospective nature, which impairs to establish definitive cause–effect relationships, and a relatively small number of enrolled subjects. It would have been interesting to evaluate whether this pharmacological effect was also observed in patients receiving concomitant antidiabetic and/or statin treatment. In addition, our findings require confirmation in non-Caucasian subjects and in patients using different N-BPs, considering that even small changes to the pharmacological structure of each N-BP may modify the ability to inhibit farnesyl-diphosphate-synthase [47]. In conclusion, the study results suggest a potentially interesting pharmacological effect of N-BPs on glucose and lipid metabolism that adds to their previously described capacity to reduce lipid deposition in the atherosclerotic plaque and to prevent vascular calcification. Further studies are warranted to confirm our observations.

## Supplementary Information

Below is the link to the electronic supplementary material.

(DOCX 21 kb)

## Data Availability

The manuscript’s guarantor affirms that this manuscript is an honest, accurate, and transparent account of the study being reported; that no important aspects of the study have been omitted; and that any discrepancies from the study as planned (and, if relevant, registered) have been explained.

## References

[1] Lorenzatti AJ, Toth PP (2020). New perspectives on atherogenic dyslipidaemia and cardiovascular disease. Eur Cardiol.

[2] Veronese N, Stubbs B, Crepaldi G, Solmi M, Cooper C, Harvey NC, Reginster JY, Rizzoli R, Civitelli R, Schofield P, Maggi S, Lamb SE (2017). Relationship between low bone mineral density and fractures with incident cardiovascular disease: a systematic review and meta-analysis. J Bone Miner Res.

[3] Golden SH, Robinson KA, Saldanha I, Anton B, Ladenson PW (2009). Clinical review: prevalence and incidence of endocrine and metabolic disorders in the United States: a comprehensive review. J Clin Endocrinol Metab.

[4] Johnell O, Kanis JA (2006). An estimate of the worldwide prevalence and disability associated with osteoporotic fractures. Osteoporos Int.

[5] Seitz S, Priemel M, von Domarus C, Beil FT, Barvencik F, Amling M, Rueger JM (2008). The second most common bone disease: a review on Paget’s disease of bone. Eur J Trauma Emerg Surg.

[6] Corral-Gudino L, Borao-Cengotita-Bengoa M, Del Pino-Montes J, Ralston S (2013). Epidemiology of Paget’s disease of bone: a systematic review and meta-analysis of secular changes. Bone.

[7] van Staa TP, Selby P, Leufkens HG, Lyles K, Sprafka JM, Cooper C (2002). Incidence and natural history of Paget’s disease of bone in England and Wales. J Bone Miner Res.

[8] Rendina D, Gennari L, De Filippo G, Merlotti D, de Campora E, Fazioli F, Scarano G, Nuti R, Strazzullo P, Mossetti G (2006). Evidence for increased clinical severity of familial and sporadic Paget’s disease of bone in Campania, southern Italy. J Bone Miner Res.

[9] Gennari L, Rendina D, Falchetti A, Merlotti D (2019). Paget’s disease of bone. Calcif Tissue Int.

[10] Rogers MJ (2003). New insights into the molecular mechanisms of action of bisphosphonates. Curr Pharm Des.

[11] Benford HL, Frith JC, Auriola S, Monkkonen J, Rogers MJ (1999). Farnesol and geranylgeraniol prevent activation of caspases by aminobisphosphonates: biochemical evidence for two distinct pharmacological classes of bisphosphonate drugs. Mol Pharmacol.

[12] Buhaescu I, Izzedine H (2007). Mevalonate pathway: a review of clinical and therapeutical implications. Clin Biochem.

[13] Rendina D, De Filippo G, Merlotti D, Di Stefano M, Succoio M, Muggianu SM, Bianciardi S, D’Elia L, Coppo E, Faraonio R, Nuti R, Strazzullo P, Gennari L (2019). Vitamin D status in Paget disease of bone and efficacy-safety profile of cholecalciferol treatment in Pagetic patients with hypovitaminosis D. Calcif Tissue Int.

[14] Rendina D, Gianfrancesco F, De Filippo G, Merlotti D, Esposito T, Mingione A, Nuti R, Strazzullo P, Mossetti G, Gennari L (2010). FSHR gene polymorphisms influence bone mineral density and bone turnover in postmenopausal women. Eur J Endocrinol.

[15] Rossini M, Adami S, Bertoldo F, Diacinti D, Gatti D, Giannini S, Giusti A, Malavolta N, Minisola S, Osella G, Pedrazzoni M, Sinigaglia L, Viapiana O, Isaia GC (2016). Guidelines for the diagnosis, prevention and management of osteoporosis. Reumatismo.

[16] Gennari L, Merlotti D, Mossetti G, Rendina D, De Paola V, Martini G, Nuti R (2009). The use of intravenous aminobisphosphonates for the treatment of Paget’s disease of bone. Mini Rev Med Chem.

[17] Mossetti G, Gennari L, Rendina D, De Filippo G, Merlotti D, De Paola V, Fusco P, Esposito T, Gianfrancesco F, Martini G, Nuti R, Strazzullo P (2008). Vitamin D receptor gene polymorphisms predict acquired resistance to clodronate treatment in patients with Paget’s disease of bone. Calcif Tissue Int.

[18] Merlotti D, Rendina D, Muscariello R, Picchioni T, Alessandri M, De Filippo G, Materozzi M, Bianciardi S, Franci MB, Lucani B, Cenci S, Strazzullo P, Nuti R, Gennari L (2020). Preventive role of vitamin D supplementation for acute phase reaction after bisphosphonate infusion in Paget’s disease. J Clin Endocrinol Metab.

[19] Tufano A, Rendina D, Conca P, Matani B, Di Minno G (2019). Hypocalcemia and hypophosphatemia after treatment with zoledronic acid in a patient with AL amyloidosis. Intern Emerg Med.

[20] Mirabello L, Troisi RJ, Savage SA (2009). Osteosarcoma incidence and survival rates from 1973 to 2004: data from the Surveillance, Epidemiology, and End Results Program. Cancer.

[21] Rendina D, De Filippo G, Ralston SH, Merlotti D, Gianfrancesco F, Esposito T, Muscariello R, Nuti R, Strazzullo P, Gennari L (2015). Clinical characteristics and evolution of giant cell tumor occurring in Paget’s disease of bone. J Bone Miner Res.

[22] Levey AS, Stevens LA, Schmid CH, Zhang YL, Castro AF, Feldman HI, Kusek JW, Eggers P, Van Lente F, Greene T, Coresh J, CKD-EPI (2009). A new equation to estimate glomerular filtration rate. Ann Intern Med.

[23] Cooper DS, Biondi B (2012). Subclinical thyroid disease. Lancet.

[24] Fillée C, Keller T, Mourad M, Brinkmann T, Ketelslegers JM (2012). Impact of vitamin D-related serum PTH reference values on the diagnosis of mild primary hyperparathyroidism, using bivariate calcium/PTH reference regions. Clin Endocrinol (Oxf).

[25] Adhyaru BB, Jacobson TA (2018). Safety and efficacy of statin therapy. Nat Rev Cardiol.

[26] Gozzetti A, Gennari L, Merlotti D, Salvadori S, De Paola V, Avanzati A, Franci B, Marchini E, Tozzi M, Campagna MS, Nuti R, Lauria F, Martini G (2008). The effects of zoledronic acid on serum lipids in multiple myeloma patients. Calcif Tissue Int.

[27] GBD 2013 Mortality and Causes of Death Collaborators (2015). Global, regional, and national age-sex specific all-cause and cause-specific mortality for 240 causes of death, 1990–2013: a systematic analysis for the Global Burden of Disease Study. Lancet.

[28] Lello S, Capozzi A, Scambia G (2015). Osteoporosis and cardiovascular disease: an update. Gynecol Endocrinol.

[29] Rendina D, De Filippo G, Muscariello R, De Palma D, Fiengo A, De Pascale F, Strazzullo P (2014). Vitamin D and cardiometabolic disorders. High Blood Press Cardiovasc Prev.

[30] Lampropoulos CE, Papaioannou I, D’Cruz DP (2012). Osteoporosis-a risk factor for cardiovascular disease?. Nat Rev Rheumatol.

[31] Sato M, Asada N, Kawano Y, Wakahashi K, Minagawa K, Kawano H, Sada A, Ikeda K, Matsui T, Katayama Y (2013). Osteocytes regulate primary lymphoid organs and fat metabolism. Cell Metab.

[32] Bone HG (2006). Nonmalignant complications of Paget’s disease. J Bone Miner Res.

[33] Gianfrancesco F, Rendina D, Merlotti D, Esposito T, Amyere M, Formicola D, Muscariello R, De Filippo G, Strazzullo P, Nuti R, Vikkula M, Gennari L (2013). Giant cell tumor occurring in familial Paget’s disease of bone: report of clinical characteristics and linkage analysis of a large Pedigree. J Bone Miner Res.

[34] Morales-Piga AA, Moya JL, Bachiller FJ, Muñoz-Malo MT, Benavides J, Abraira V (2000). Assessment of cardiac function by echocardiography in Paget’s disease of bone. Clin Exp Rheumatol.

[35] Arnalich F, Plaza I, Sobrino JA, Oliver J, Barbado J, Peña JM, Vazquez JJ (1984). Cardiac size and function in Paget’s disease of bone. Int J Cardiol.

[36] Bevilacqua M, Dominguez LJ, Rosini S, Barbagallo M (2005). Bisphosphonates and atherosclerosis: why?. Lupus.

[37] Persy V, De Broe M, Ketteler M (2006). Bisphosphonates prevent experimental vascular calcification: treat the bone to cure the vessels?. Kidney Int.

[38] Guney E, Kisakol G, Ozgen AG, Yilmaz C, Kabalak T (2008). Effects of bisphosphonates on lipid metabolism. Neuro Endocrinol Lett.

[39] Gonnelli S, Caffarelli C, Tanzilli L, Pondrelli C, Lucani B, Franci BM, Nuti R (2014). Effects of intravenous zoledronate and ibandronate on carotid intima-media thickness, lipids and FGF-23 in postmenopausal osteoporotic women. Bone.

[40] Passeri E, Mazzaccaro D, Sansoni V, Perego S, Nano G, Verdelli C, Lombardi G, Corbetta S (2019). Effects of 12-months treatment with zoledronate or teriparatide on intima-media thickness of carotid artery in women with postmenopausal osteoporosis: a pilot study. Int J Immunopathol Pharmacol.

[41] Sing CW, Wong AY, Kiel DP, Cheung EY, Lam JK, Cheung TT, Chan EW, Kung AW, Wong IC, Cheung CL (2018). Association of alendronate and risk of cardiovascular events in patients with hip fracture. J Bone Miner Res.

[42] Vestergaard P (2012). Acute myocardial infarction and atherosclerosis of the coronary arteries in patients treated with drugs against osteoporosis: calcium in the vessels and not the bones?. Calcif Tissue Int.

[43] Lu P-Y, Hsieh C-F, Tsai Y-W, Huang W-F (2011). Alendronate and raloxifene use related to cardiovascular diseases: differentiation by different dosing regimens of alendronate. Clin Ther.

[44] Kang JH, Keller JJ, Lin HC (2012). A population-based 2-year follow-up study on the relationship between bisphosphonates and the risk of stroke. Osteoporos Int.

[45] Rendina D, De Filippo G, Strazzullo P (2010). Should vitamin D status be assessed in patients with congestive heart failure?. Nutr Metab Cardiovasc Dis.

[46] Greco EA, Lenzi A, Migliaccio S (2019). Role of hypovitaminosis D in the pathogenesis of obesity-induced insulin resistance. Nutrients.

[47] Dunford JE, Thompson K, Coxon FP, Luckman SP, Hahn FM, Poulter CD, Ebetino FH, Rogers MJ (2001). Structure–activity relationships for inhibition of farnesyldiphosphate synthase in vitro and inhibition of bone resorption in vivo by nitrogen-containing bisphosphonates. J Pharmacol Exp Ther.

