# Supplementary material for: Effects of Bisphosphonate Treatment on Circulating Lipid and Glucose Levels in Patients with Metabolic Bone Disorders
Source: Calcif Tissue Int. 2021 Feb 9;108(6):757–63. doi: 10.1007/s00223-021-00811-w (PMC8166716; doi:10.1007/s00223-021-00811-w)
Supplement: Supplementary file 1 — (DOCX 21 kb) [file 223_2021_811_MOESM1_ESM.docx]

**Additional table 1**

**Absolute and percentage variations of biochemical parameters from baseline to one month and six months after bisphosphonate treatment in the three study cohorts**

|  |  |  |  |  |  |  |  |  |  |  |  |  |  |  |
| --- | --- | --- | --- | --- | --- | --- | --- | --- | --- | --- | --- | --- | --- | --- |
|  | **PDB-Zol** | | | |  | **PDB-Clo** | | | |  | **Op-Zol** | | | |
|  | **T1-Abs** | **T1-%** | **T6-Abs** | **T6-%** |  | **T1-Abs** | **T1-%** | **T6-Abs** | **T6-%** |  | **T1-Abs** | **T1-%** | **T6-Abs** | **T6-%** |
| **ΔCrea (μmol/l)** | 1.50±0.19 | 1.88±0.23 | 0.70±0.09 | 0.88±0.11 |  | 2.00±0.21 | 2.47±0.26 | -1.30±0.17 | -1.61±0.21 |  | 1.20±0.18 | 1.48±0.22 | -1.00±0.11 | -1.24±0.13 |
| **ΔGlu (mmol/l)** | -0.21±0.04^a^ | -4.05±0.78 ^a^ | -0.28±0.05 ^a^ | -5.39±0.96 ^a^ |  | 0.18±0.03 | 3.44±0.57 | 0.04±0.01 | 0.76±0.19 |  | -0.28±0.05 ^a^ | -5.33±0.95 ^a^ | -0.35±0.06 ^a^ | -6.67±1.14 ^a^ |
| **ΔtCa (mmol/l)** | -0.08±0.01 | -3.38±0.42 | -0.05±0.01 | -2.11±0.42 |  | -0.12±0.02 | -4.98±0.83 | -0.01±0.01 | -0.41±0.12 |  | -0.13±0.02 | -5.56±0.85 | -0.02±0.01 | -0.85±0.18 |
| **Δt-Chol (mmol/l)** | -0.21±0.04 ^a^ | -4.12±0.04 ^a^ | -0.39±0.05 ^a^ | -7.65±0.98 ^a^ |  | -0.11±0.02 | -2.08±0.38 | -0.06±0.01 | -1.13±0.18 |  | -0.23±0.04 ^a^ | -4.52±0.79 ^a^ | -0.37±0.06 ^a^ | -7.27±1.18 ^a^ |
| **ΔHDL-chol (mmol/l)** | -0.01±0.01 | -0.76±0.12 | -0.01±0.01 | -0.76±0.16 |  | -0.10±0.01 | -6.71±0.61 | -0.10±0.01 | -6.71±0.63 |  | -0.01±0.01 | -0.66±0.07 | -0.01±0.01 | -0.66±0.06 |
| **ΔLDL-chol (mmol/l)** | -0.12±0.02 ^a^ | -3.87±0.64 ^a^ | -0.27±0.04 ^a^ | -8.71±1.29 ^a^ |  | 0.01±0.01 | 0.32±0.08 | 0.06±0.01 | 1.93±0.28 |  | -0.16±0.03 ^a^ | -5.48±1.02 ^a^ | -0.26±0.05 ^a^ | -8.90±1.71 ^a^ |
| **ΔTG (mmol/l)** | -0.17±0.03 | -11.41±2.01 | -0.24±0.04 ^a^ | -16.11±2.68 ^a^ |  | -0.05±0.01 | -3.27±0.54 | -0.03±0.01 | -1.96±0.31 |  | -0.15±0.03 | -10.27±2.05 | -0.22±0.04 ^a^ | -15.07±2.74 ^a^ |
| **Δ25OHD (nmol/l)** | -1.90±0.37 | -2.25±0.44 | -0.62±0.11 | -0.71±0.13 |  | -0.70±0.12 | -0.79±0.14 | -2.00±0.39 | -2.24±0.44 |  | -1.70±0.28 | -2.00±0.32 | -3.50±0.61 | -4.11±0.72 |
| **ΔALP (%)** | -68±19 ^a^ | -43.9±12.3 ^a^ | -77±18 ^a^ | -49.7±11.6 ^a^ |  | -34±7 ^a^ | -23.0±4.7 ^a^ | -65±14 ^a^ | -43.9±9.45 ^a^ |  | -8±1.4 | -10.7±1.9 | -10±1.8 | -13.3±2.39 |

Data are expressed as mean ± standard deviation; PDB-Zol: patients with Paget’s disease of bone treated with zoledronic acid; PDB-Clo: patients with Paget’s disease of bone treated with clodronic acid; Op-Zol: patients with osteoporosis treated with zoledronic acid; T1-Abs: absolute variations one month after zoledronic or clodronic acid treatment; T1-%: percentage variations one month after zoledronic or clodronic acid treatment; T6-Abs: absolute variations six months after zoledronic or clodronic acid treatment; T6-%: percentage variations six months after zoledronic or clodronic acid treatment; **Δ**Crea: creatinine variation from baseline; **Δ**Glu: glycemia variation from baseline; **Δ**tCa: total calcium variation from baseline; **Δ**t-Chol: total cholesterol variation from baseline; **Δ**HDL-chol: high density lipoprotein-cholesterol variation from baseline; **Δ**LDL-chol: low density lipoproteins-cholesterol variation from baseline; **Δ**TG: triglycerides variation from baseline; **Δ**25OHD: 25OH vitamin D variation from baseline; **Δ**ALP: total alkaline phosphatase variation from baseline; ^a^: significantly different compared to baseline (p<0.05; T-Test for paired samples).
